# A novel near-infrared EGFR targeting probe for metastatic lymph node imaging in preclinical mouse models

**DOI:** 10.1186/s12951-023-02101-z

**Published:** 2023-09-22

**Authors:** Diya Xie, Yunlong Li, Jiahong Shi, Yao Ping Zhu, Yiqing Wang, Christopher J. Butch, Zhiyong Wang

**Affiliations:** 1 Department of Oral and Maxillofacial Surgery, Affiliated Hospital of Medical School, Nanjing Stomatological Hospital, Nanjing University, Nanjing, China; 2 Department of Biomedical Engineering, College of Engineering and Applied Sciences, Nanjing University, Nanjing, China; 3 State Key Laboratory of Analytical Chemistry for Life Science, Nanjing University, Nanjing, China; 4 Department of Periodontics, Affiliated Hospital of Medical School, Nanjing Stomatological Hospital, Nanjing University, Nanjing, China

**Keywords:** Oral squamous cell carcinoma (OSCC), Lymph node metastasis, Fluorescence-guided surgery, Near-infrared probe, EGFR targeting

## Abstract

For the treatment of patients with oral squamous cell carcinoma (OSCC), the imaging of cervical lymph nodes and the evaluation of metastastic progression are of great significance. In recent years, the development of new non-radioactive lymph node tracers has been an area of intense research. Here, we report the synthesis, good biocompatibility, and in vivo evaluation of a new small molecule near-infrared (NIR) fluorescence probe by the conjugation of Lapatinib to S0456 (LP-S). We show that like Lapatinib, LP-S binds to the epidermal growth factor receptor (EGFR) resulting in high quality fluorescence imaging of metastatic lymph nodes in OSCC mouse models. After local injection of LP-S into the tumor, the lymphatic drainage pathway and lymph nodes can be clearly identified by NIR fluorescence imaging. Further, the LP-S probe shows higher contrast and longer retention in metastatic lymph nodes, allowing them to be differentiated from normal lymph nodes, and affording a new choice for fluorescence-guided surgery.

**Graphical abstract:**

**Figure:**
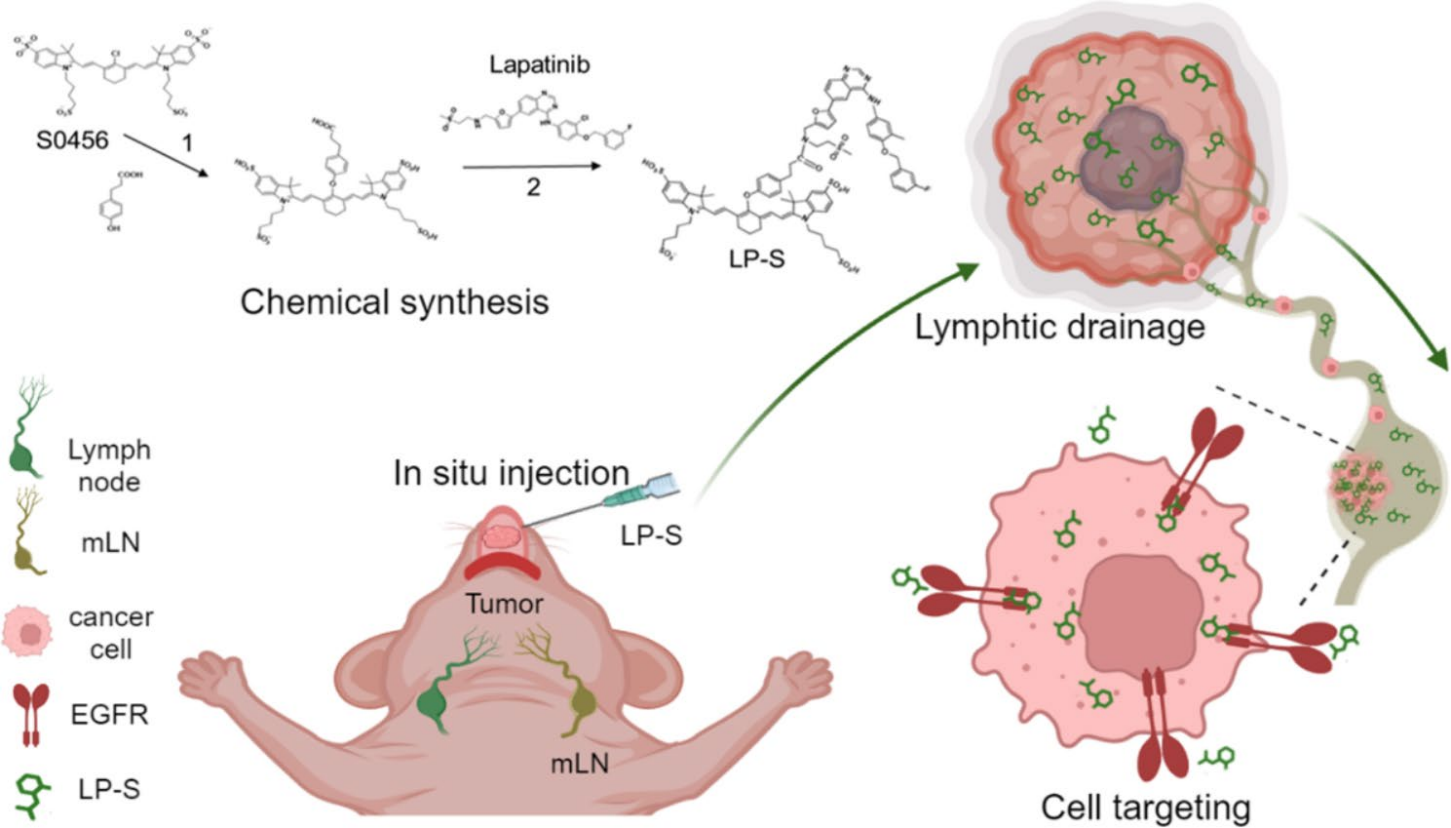
Scheme. Chemical synthesis and application of EGFR targeting probe LP-S for imaging of metastatic lymph nodes (mLNs) in OSCC

**Supplementary Information:**

The online version contains supplementary material available at 10.1186/s12951-023-02101-z.

## Introduction

For patients with oral squamous cell carcinoma (OSCC), the most common route of metastatic progression is by limbic drainage to the first cervical sentinel lymph node (SLN) [1–4]. Metastasis to the cervical lymph node (LN) is regarded as one of the most important prognostic markers in OSSC treatment because the presence of a single metastatic lymph node (mLN) is associated with a decrease of up to 50% in the 5-year survival rate [5, 6]. Therefore, identification of cervical LN metastasis is critical for the staging diagnosis, treatment plan and prognostic evaluation of OSCC patients [7–11]. To meet this medical need, the development of dye molecules which can specifically identify cancer cells both preoperatively and intra-surgically is an attractive avenue of research.

Traditional techniques such as ultrasonography, CT, MRI, and PET imaging have been commonly used for preoperative localization of cervical mLNs [12–15]. However, identification of early-stage lymph node metastases using these technologies is difficult if not impossible. Further, none of these techniques can be employed for real-time guidance during surgery. As an alternative to these techniques, a large and growing body of research has shown that near-infrared (NIR) fluorescence-guidance is able to distinguish between normal and pathological structures both pre- and intra-operatively [16–19]. The most common clinically used NIR dye, indocyanine green (ICG) was licensed for human use in 1956, and has been employed for LN imaging in various cancer treatments for decades [20–24]. After injection, accumulation of ICG in an LN can indicate both the need for surgical resection and guide the complete LN removal. Combined with additional subsequent pathological examination of resected LNs this approach provides an effective means to detect the presence and possible metastases of cancer, providing guidance for correct and effective treatment.

Although ICG enjoys widespread clinical use for imaging tumor lesions and LNs, it does have deficiencies which can be improved upon to yield better patient outcomes. ICG accumulation in tumors and LNs is mostly passive, often leading to false positive results due to its lack of targeting [25–27]. In our previous research, we found using ICG to identify cervical LNs was quite accurate, but distinguishing mLNs had a false positive rate of 72.83% [28]. Thus, there is still significant medical need for fluorescent dyes which specifically target metastatic tumor cells to distinguish mLN from normal LNs.

The design of tumor specific NIR probes by conjugating a ligand with high affinity to an overexpressed receptor is a proven strategy to increase the accumulation of contrast agents in malignant lesions and improve imaging performance. OSCC cells usually overexpress epidermal growth factor receptor (EGFR), which is a promising target for specific binding [29–33]. In this study, we developed an EGFR targeting fluorescent dye (LP-S), by coupling the small molecule EGFR inhibitor Lapatinib with NIR fluorescent dye S0456. We demonstrate that LP-S has excellent biocompatibility and optical properties in vitro and is highly effective in vivo for tumor imaging and lymphatic drainage pathway tracking. Due to the EGFR binding affinity of Lapatinib, LP-S specifically accumulates in OSCC cells with high EGFR expression and provides better contrast and longer accumulation in mLNs. Based on these results, we conclude that LP-S is a promising probe for NIR imaging of cancers with high EGFR expression in future clinical applications.

## Materials and methods

ICG was purchased from Yichang Pharmaceutical Co., Ltd (Dandong, China). CCK-8, PBS, and DMEM were purchased from KeyGEN BioTECH (Jiangsu, China). Fetal bovine serum (FBS) was purchased from Biological Industries (Israel). Real-IGS system, sponsored by Nanjing Nuoyuan Medical Devices Co., Ltd. (Nanjing China), was used for fluorescence imaging. Imaging experiments were also performed using IVIS Image (PerkinElmer, Inc., USA).

### Synthesis and characterization of LP-S

#### Synthesis of S0456-HPPA

S0456 (0.100 g, 0.113 mmol) was dissolved in 2 mL of distilled dimethyl formamide (DMF) and the temperature raised to 40 ℃ in an argon atmosphere to dissolve. 4-Hydroxyphenylpropionic acid (0.125 g, 0.755 mmol) and sodium hydride (0.036 g, 1.51 mmol) were added to a new round bottomed flask, and 2mL of DMF was added and raised to room temperature for 10 min. The HPPA and NaOH solution was then slowly added to the S0456 using a syringe. The reaction was stirred for 1 h, and then quenched and an aliquot sample was collected in a mixture of ether and 1% acetic acid, centrifuged, decanted, and the residue dissolved in water, analyzed by LCMS. The reaction mixture was treated with 20 mL of 1:1 v/v acetonitrile: ether solution and 50 µL of acetic acid. The mixture was quenched and left overnight at 4 ℃ to allow the product to precipitate. The crude material was collected through vacuum filtration, washed with ether, and the product was dried under vacuum.

Subsequently, the product was further purified on a C-18 column using a 10–50% acetonitrile buffer system through reverse phase preparative HPLC. The fraction was determined using LC-MS. The sample was loaded into 0.1 M trifluoroacetic acid. The collected fractions containing pure products were merged, concentrated, and subjected to freeze-drying, which was repeated three times. Then, ion exchange was carried out to form sodium salts. The final step involved performing freeze-drying to obtain the product as S0456 carboxylate (0.0815 g, 0.0848 mmol, 75.0% yield). 1 H nuclear magnetic resonance (500 MHz, D 2O) δ 8.11 (d, J = 8.9 Hz, 1 H), 7.86 (d, J = 8.6 Hz, 1 H), 7.84–7.79 (m, 1 H), 7.71–7.64 (m, 4 H), 7.59 (s, 3 H) ), 7.14 (t, J = 8.6 Hz, 4 H), 6.94–6.80 (m, 2 H), 5.92 (d, J = 14.0 Hz, 2 H), 3.92–3.77 (m, 4 H), 2.81 (t, J = 6.8 Hz, 5 H), 2.77–2.68 (m, 2 H), 2.54–2.41 (m, 4 H), 2.37 (t, J = 7.8 Hz, 2 H), 1.80 (s, 2 H), 1.76–1.66 (m, 9 H) ), 1.32 (s, 5 H), 1.08 (d, J = 3.1 Hz, 13 H)。 Calculated value of LC-MS (ES -), C47H56N2O15S4: 1017.25635; LC-MS measured values [m/z] -: 1016.04 (MH), 507.34 (M-2 H/2), 337.86 (M-3 H/3); HRMS (ES -), found [m/z] -: 1016.2496 (MH), 507.1202 (M-2 H/2), 337.7436 (M-3 H/3); UV-Vis λ Max = 775.53 (in LC-MS DAD, methanol/buffer was used).

#### Synthesis of S0456-HPPA-NHS

S0456-HPPA (0.070 g, 0.0728 mmol) was dissolved in 2 mL of DMF and heated to 45 ℃. N, N, N’, N’-tetramethyl-O-(N-succinimidyl) tetrafluoroborate urea (0.0439 g, 0.146 mmol) and DIEA (0.113 g, 8.74 mmol) were combined and added to the solution. The reaction was allowed to proceed for 30 min with stirring. Subsequently, 10 mL of ether was added and the reaction mixture was stored at 4 °C overnight for precipitation.

The crude material was filtered and collected, followed by washing with ether. The obtained NHS esters (0.073 g, 0.069 mmol, 95.0% yield) were vacuum-dried. Due to the high reactivity of NHS esters, the purity was confirmed by dissolving trace amounts in 0.01% butyl amine. An aliquot sample was quenched, and butyl amide derivatives were determined using LC-MS for verification. Only S0456-NHS esters with a purity of>95% were used in the study. LC-MS (ES-) quenching of butyl amide, calculated as C51H65N3O14S4:1071.33, LC-MS measured values 1071.52 (M-H), 534.98 (M-2 H/2), 356.49 (M -3 H/3).

#### Synthesis of LP-S

Place S0456-HPPA-NHS (1.15 g, 1 mmol) into a round bottom flask and dissolve it in 20ml DMSO. Then, Lapatinib (0.58 g, 1 mmol) and DIEA (258 mg, 2mmol) were added at 37 ℃ and stir for 24 h. Precipitate in acetone solution, filter and vacuum dry. 1.6 g was obtained, and the product was purified by preparative liquid chromatography to obtain product LP-S with a purity of > 98%. LC-MS (ES-) calculated as C80H89N7O17S5ClF:1559.83, LC-MS measured values 1559.98 (M-H), 778.42 (M-2 H/2), 519.23 (M-3 H/3); UV-Vis λ Max = 781 nm (in LC-MS DAD, methanol/buffer was used).

### Molecular docking

Protein structures for molecular docking were obtained from the RCSB Protein Data Bank. For EGFR, PDB-ID 4G5J was selected along with 3PP0 for HER2. Both structures contain a small molecule inhibitor in the Lapatinib binding site. The protein structure was prepared in Chimera by removing the ligand and running Dock Prep and subsequent structure minimization. Ligands were prepared by first drawing the dye conjugate in ChemDraw followed by protonation and UFF minimization using RDKit. Charges were calculated using mmff94 in Openbabel. Docking was performed using SMINA with seed 0, autobox_ligand parameter centered on the original ligand, autobox_add parameter is 15, exhaustiveness parameter is 24.

### Animals and cells

Balb/c male mice (6 weeks old, 18–20 g) were purchased from the Huachuang Xinnuo Pharmaceutical Technology Co., Ltd (Jiangsu, China). All the animal researches were performed in compliance with protocols approved by the animal protection committee of Nanjing University. This study was approved by the medical ethics committee of the Institute Affiliated Stomatology Hospital, Nanjing University Medical School.

The oral squamous cell carcinoma (OSCC) cell lines were purchased from the Type Culture Collection of the Chinese Academy of Sciences (Shanghai, P. R. China). CAL27, SCC9 and HSC3 cells were cultured in DMEM medium with 10% FBS and 1% penicillin-streptomycin and incubated in a humidified incubator at 37 °C with 5% CO2.

### Cellular uptake

For cell uptake measurements, 5 × 10^5^ cells were plated in a twelve well culture plate and grown overnight. Fresh medium combined with 50 μm NIR fluorescent dye LP-S was added to each well the following day. Flowing incubation at 37 ° C for 1, 2 and 4 h, the cells were washed 3 times with PBS to remove any unbound fluorescent probe. Cells were then observed using the NIR imaging-guided instrument (REAL-IGS) and fluorescence microscopy. ImageJ was used to analyze intracellular fluorescence intensity.

### Body distribution

The mouse tumor model (CAL27 tumor xenograft in BALB/c mice) was utilized for the in vivo bio-distribution assessment of LP-S. After reaching a tumor volume of 300 mm^3^, mice were pooled and injected intravenously with LP-S and ICG (0.1 mg/ml, 100ul). Following injection, mice were imaged using an IVIS system and the REAL-IGS system at an excitation wavelength of 780 nm and an emission wavelengths of 820 nm. Mice were sacrificed after 48 h, and major organs and the tumor tissues were dissected and washed with PBS and subsequently imaged under the same condition.

### Models of LN metastases

In order to establish the LN metastasis model, CAL27 cells (5 × 10^5^ in 50 µl of PBS) were injected into the tongue or left hock area of BALB/c mice. LP-S in PBS (25ug/ml, 50ul) was injected into the tongue or footpad of mouse model. After fluorescence intensities were measured by a hand-held NIR fluorescence spectrometer (MaYa 2000 Pro, Ocean Optics, Dunedin, FL, USA), the fluorescence imaging was performed and the fluorescence-positive LNs were removed for pathological examination.

### Data analysis

Differences between different groups were calculated with two-tailed Student’s t-test. All of the data were displayed as mean ± standard derivation (SD). A value of p < 0.05 was considered statistically significant and was indicated by asterisks in the figures.

## Results

### Synthesis and characteristics of LP-S

**Fig. 1:**
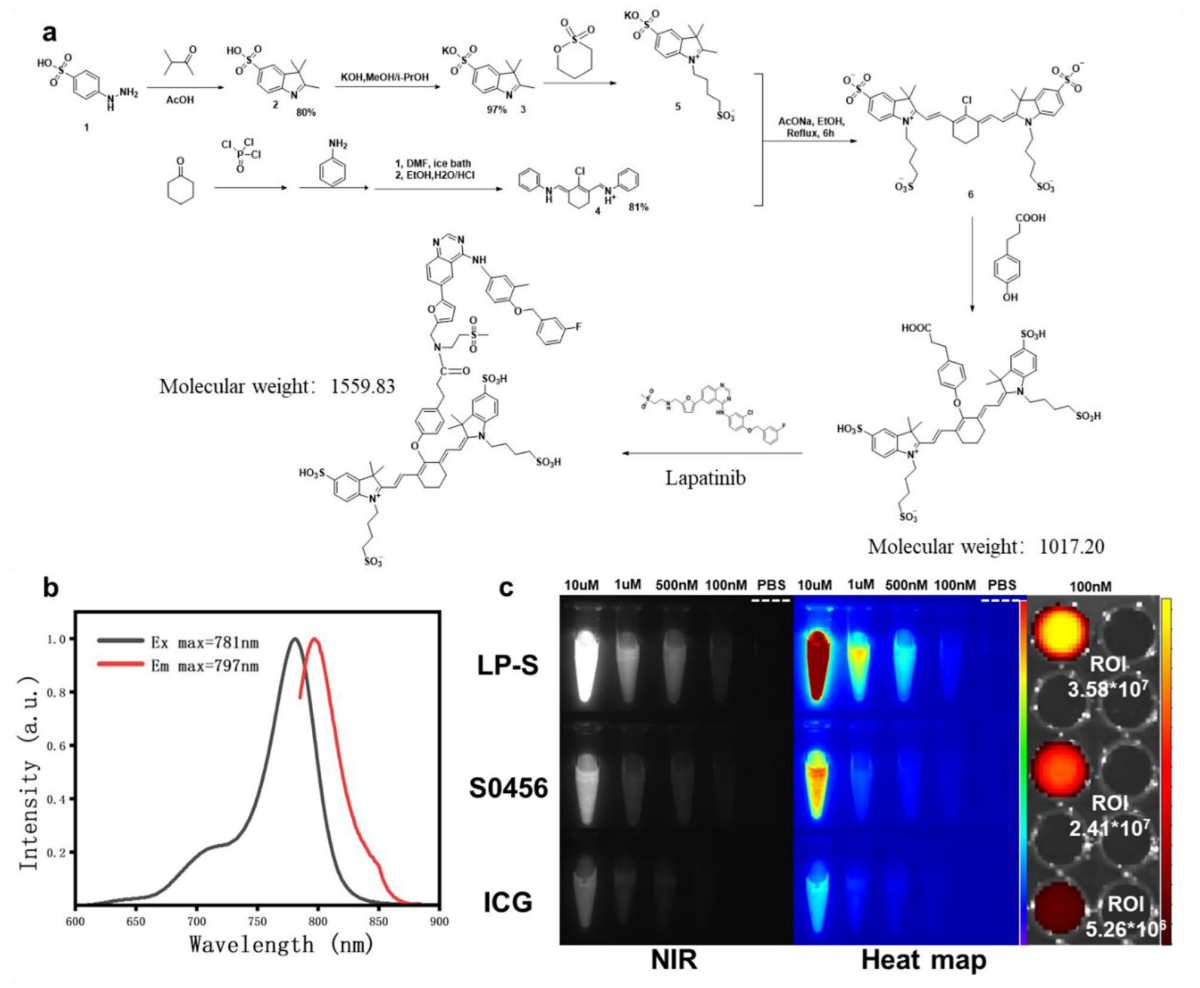
(**a**) The design and synthesis scheme of LP-S. (**b**) The excitation (Ex) and emission (Em) spectra of LP-S in PBS. (c) In vitro NIR fluorescence image and heat maps of LP-S, S0456 and ICG in PBS with different concentrations (10 μm, 1 μm, 500nM, 100nM and PBS). Overlay fluorescence image over white light image of solid black well plate containing 100nM of LP-S, S0456 and ICG in PBS. ROI = Region of interest

The designs and synthesis of LP-S is presented in Fig. 1a. Briefly, p-hydroxy phenylpropanoic acid (HPPA) added to the meso-chloride of S0456 by dropwise addition under heavy stirring to avoid biproduct formation. After purification, the carboxylic acid group of HPPA was activated by incorporation of an NHS leaving group. After additional purification, S0456-HPPA-NHS was reacted with the secondary amine of Lapatinib in DMSO to from a secondary amide linkage. The product was precipitated with acetone and further purified by liquid chromatography yielding LP-S with 98% purity (as depicted in Figure S1). The use of HPPA as the linking moiety serves two purposes. First the aromatic ring contributes to the delocalized electron framework of the NIR dye somewhat enhancing the fluorescent properties, and second as a semi rigid linker, it creates the necessary spacing to ensure Lapatinib is still able to bind within EGFR. Synthesized LP-S has maximum excitation and emission wavelengths at 781 and 797 nm, respectively slightly differing from the parent dye (Fig. 1b). LP-S water solubility was increased compared to Lapatinib (Figure S2) and its fluorescence intensity was significantly higher than either ICG or S0456 at equimolar concentrations (as illustrated in Fig. 1c). Further analysis showed the quantum yield of LP-S was 9.45% much higher than the 0.48% of ICG at the same conditions (Figure S3a). The photostability of LP-S was also much higher than ICG as seen in Figure S3b and S3c.

**Fig. 2:**
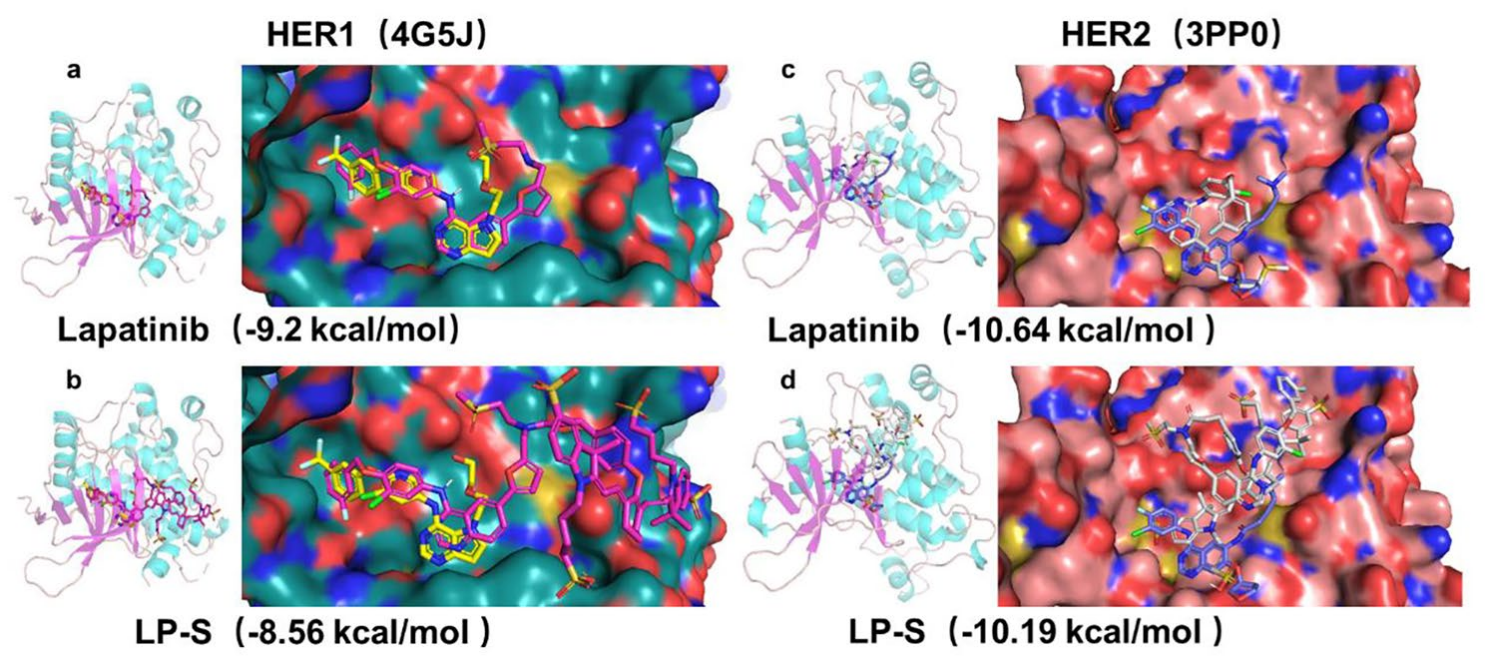
Study of molecular docking. (**a**) HER1 and Lapatinib with binding energy − 9.2 kcal/mol. (**b**) HER1 and LP-S with binding energy − 8.56 kcal/mol. (**c**) HER2 and Lapatinib with binding energy − 10.64 kcal/mol. (**d**) HER2 and LP-S with binding energy − 10.19 kcal/mol

*In silico* evaluation of LP-S binding to EGFR and HER2 and comparison to Lapatinib was conducted using SMINA. The crystal structures of these two receptors in complex with small molecule inhibitors are available in the Protein Data Bank in high resolution (EGFR/HER1: 4G5J, HER2: 3PP0). When Lapatinib and LP-S were docked in place of the co-crystalized ligand their binding modes and binding energies were very similar in both EGFR and HER2 binding pockets (Fig. 2).

Finally, we evaluated the cytotoxicity of LP-S using the CCK-8 assay. The viability of OSCC cells incubated with LP-S measured at various concentrations(Figure S4). At a concentration of 10 μm LP-S cell viability remained high at 80% compared to just 30% in the Lapatinib group. For the purposes of diagnostic targeting, this increased biocompatibility is beneficial as it indicates a lower likelihood of undesirable off target effects.

### Targeting ability of LP-S in EGFR high expression cells

**Fig. 3:**
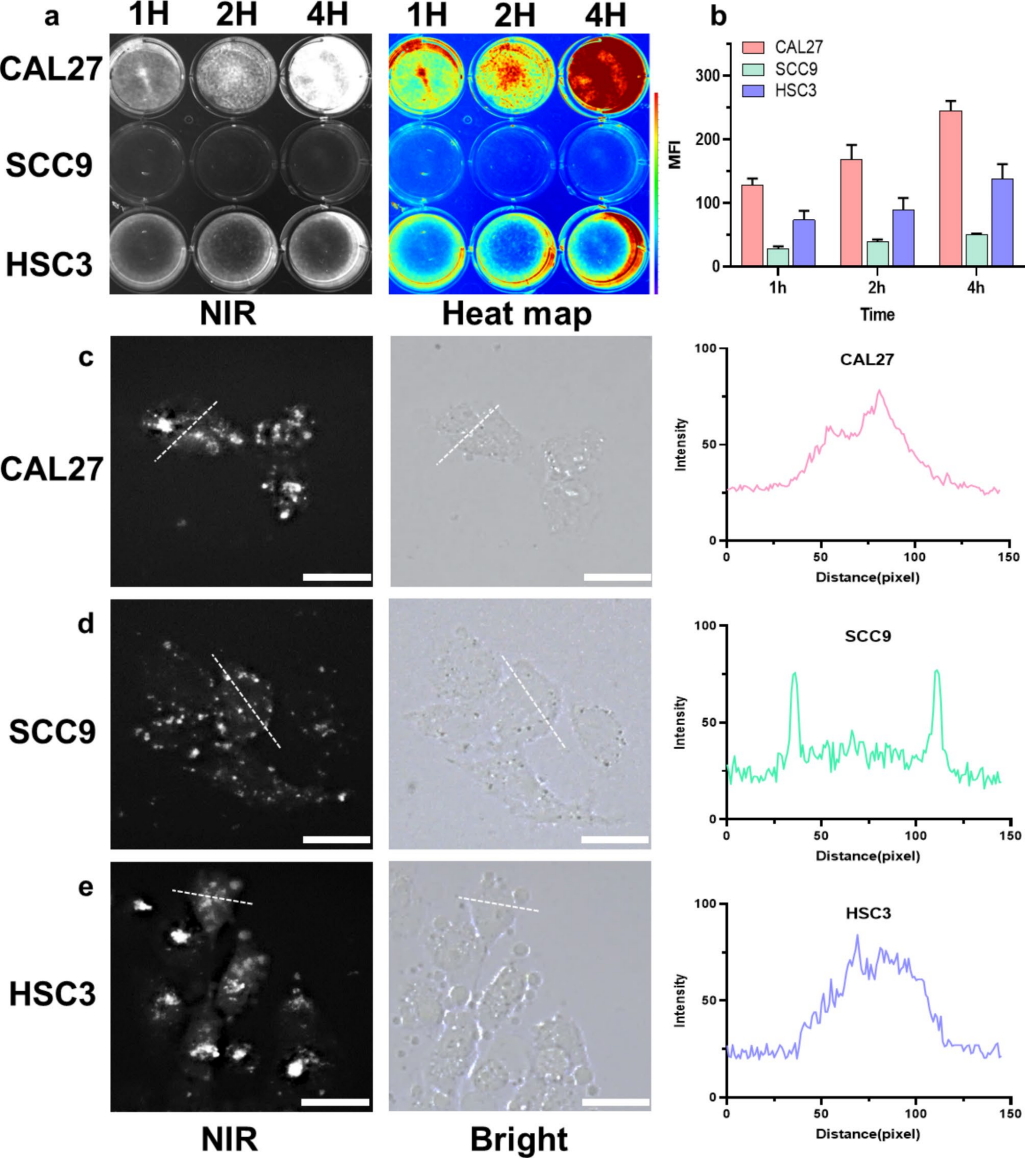
In vitro targeting ability of LP-S in OSCC cells. (**a**) NIR imaging and heat maps of OSCC cells after co-incubation with LP-S after 1 h, 2 and 4 h. (**b**) Quantitative analysis of fluorescence intensity shown in (**a**) (mean ± SD, n = 3, MFI: mean fluorescence intensity). The local magnified NIR and white images of fluorescence microscopy in CAL27 (**c**), SCC9 (**d**) and HSC3 (**e**) cells. ImageJ software analysis showing a plot of gray value versus distance across the dotted line. (Scale bar: 30 μm)

To investigate the targeted affinity of LP-S towards EGFR-expressing cancer cells, we employed three OSCC cell lines characterized by varying levels of EGFR expression. As seen in Figure S5, Cal27 and HSC3 cells have increased EGFR expression, while SCC9 cells do not. LP-S was co-incubated with the OSCC cell lines at a concentration of 50 μm for 1 h, 2 h, and 4 h. The cells were washed and imaged by fluorescent plate reader as seen in Fig. 3a. Cal27 and HSC3 cells, with their higher EGFR expression, showed more pronounced fluorescence demonstrating a heightened degree of LP-S binding. A quantitative analysis of fluorescence intensity showed the uptake of LP-S was time-dependent over the intervals investigated (Fig. 3b). Fluorescence microscopy showed a high amount of cytoplasmic LP-S in CAL27 and HSC3 cells after the 4-hour incubation period. Notably, cytoplasmic LP-S was not observed in SCC9 cells, with LP-S instead adhering to the cell surface. Quantitative ImageJ analysis reflected this difference with cellular cross sections of SCC9 cells showing two regions of fluorescence intensity at the cell periphery, as compared to the more homogeneous fluorescent distribution in CAL27 and HSC3 cells (Fig. 3c and d, and 3e). Collectively, these findings underscore LP-S targeting and internalization within OSCC cells with elevated EGFR expression.

**Fig. 4:**
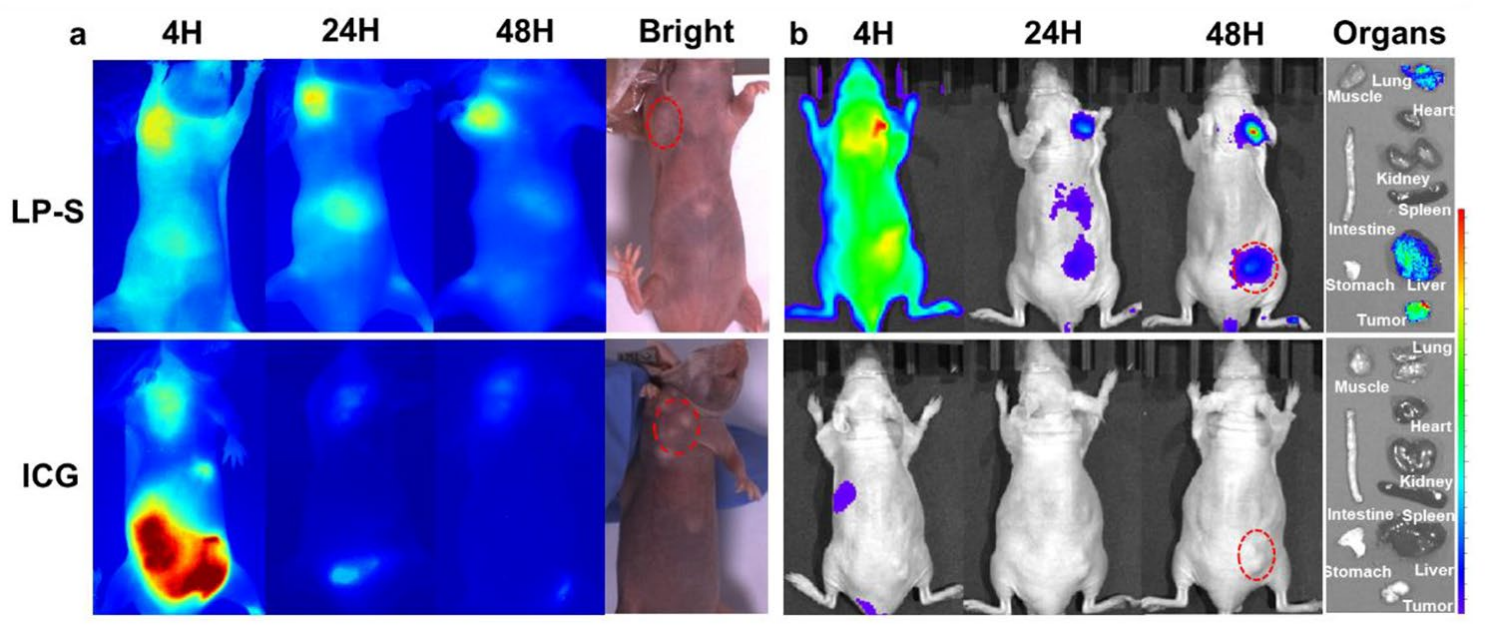
In vivo specificity and bio-distribution of LP-S in mice bearing CAL27 cells. (**a**) NIR imaging of LP-S or ICG in mice with axillary subcutaneous tumor (red circle) at different time points after administration (4 h, 24 and 48 h). (**b**) Overlay of fluorescence images over white light images of LPS or ICG in mice with subcutaneous tumors on the back (red circle) at different time points after administration (4 h, 24 and 48 h), as well as the tissue bio-distribution after 48 h of administration

Next, we evaluated the specificity of LP-S targeting within mice bearing transplanted tumors marked by high EGFR expression (Fig. 4). In live mouse experiments, LP-S, dissolved in PBS, was administered to mice with CAL27 tumors through a tail-vein injection. We compared it with the clinically approved NIR dye ICG, using the same concentration of 0.1 mg/ml. The results showed that ICG’s NIR fluorescence in the mice was weak, making it difficult to identify the tumor. However, LP-S displayed a strong fluorescence intensity suitable for NIR imaging. Both ICG and LP-S produced strong signals in the liver and intestines during real-time imaging, suggesting they follow similar metabolic pathways. Interestingly, LP-S stayed longer in the body and accumulated more in the tumor tissue, while ICG was mostly gone within 24 h. After 48 h the mice were sacrificed and fluorescence imaging of organs showed significant LP-S accumulation in the tumor with a small amount in the lungs and liver. These findings suggest that LP-S performs better than ICG as an optical tracer.

### Detection of LNs in vivo by NIR fluorescence imaging

**Fig. 5:**
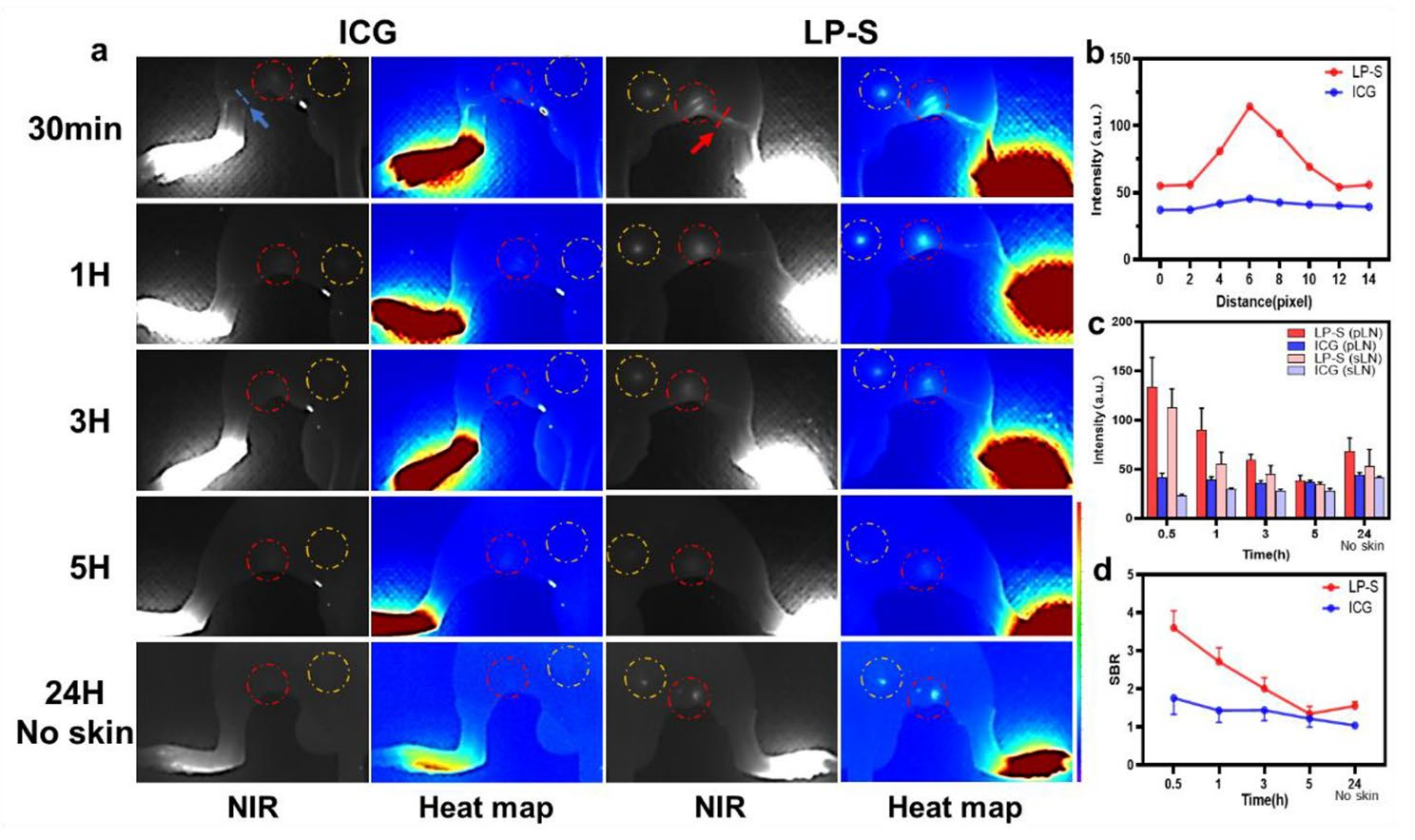
LP-S enables high contrast LNs imaging in vivo. (**a**) Representative NIR fluorescence images and heat maps of sciatic and popliteal LNs at different time points after local injection of LP-S or ICG (25ug/ml, 50ul). Yellow circle labeling of sciatic lymph node (sLN) and red circle labeling of popliteal lymph node (pLN). (**b**) Cross-sectional fluorescence intensity profiles of LP-S or ICG along the line in images after 30 min of injection. (**c**) Quantitative analysis of fluorescence intensity in sLNs and pLNs shown in (**a**) (mean ± SD, n = 3). (**d**) Signal to background ratio (SBR) of pLNs shown in (**a**) (mean ± SD, n = 3)

For lymph node (LN) imaging, we studied how well LP-S traveled through the lymphatic system in a mouse model. We injected LP-S or ICG (25ug/ml, 50ul) into the mouse’s hind footpad and then tracked how the tracer moved and how long it stayed using wide field fluorescence imaging. As shown in Fig. 5a, LP-S moved quickly to both the popliteal LNs (pLNs) and sciatic LNs (sLNs) within 30 min. This movement through the lymphatic drainage was clearly visible against the normal tissue, as shown in Fig. 5b. The fluorescence intensity of LP-S in pLNs and sLNs started to decrease after 30 min, becoming similar to the skin’s fluorescence by 5 h, as seen in Fig. 5c. The peak signal-to-background ratio (SBR) for LP-S in pLNs was 3.60 ± 0.45 at 30 min, which decreased to 1.34 ± 0.20 by 5 h by external imaging, as shown in Fig. 5d. However, 24 h after injection and after the mouse was sacrificed and the skin removed, the fluorescence signals of LP-S within pLNs were strong enough to be seen, with an SBR of 1.55 ± 0.11. This increased SBR at 24 h is believed to be due to the tracer moving into the skin, increasing the background fluorescence in wide field imaging. In comparison, ICG didn’t show clear lymphatic flow and had minimal accumulation in LNs. Also, 24 h and skin removal, its SBR reduced to 1.04 ± 0.09. Overall, these results demonstrate that LP-S is better than ICG for imaging lymphatic drainage and locating LNs.

**Fig. 6:**
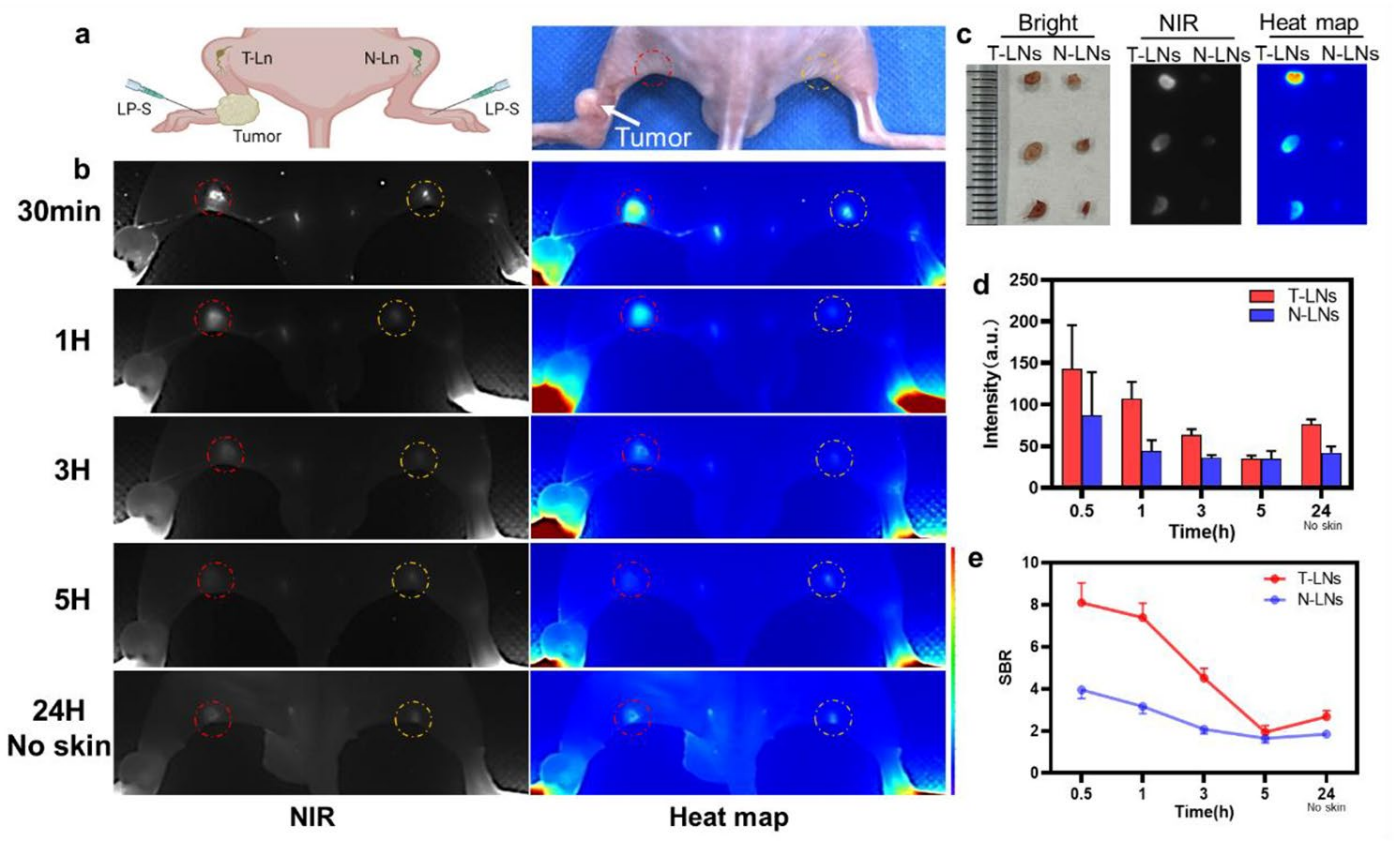
LP-S efficiently targeted metastatic LNs in vivo. (**a**) Schematic of LN tumor metastasis detection after local injection of LP-S. (**b**) Representative NIR fluorescence images and heat maps of popliteal tumor metastatic lymph nodes (T-LNs, red circle) and contralateral normal lymph nodes (N-LNs, yellow circle) at different time points after local injection of LP-S (25ug/ml, 50ul). (**c**) Fluorescence images of excised T-LNs and N-LNs after 24 h of injection (n = 3 mice/group). (**d**) Quantitative analysis of fluorescence intensity in T-LNs and N-LNs shown in (**b**) (mean ± SD, n = 3). (**e**) Signal to background ratio (SBR) of T-LNs and N-LNs shown in (**b**) (mean ± SD, n = 3)

We next evaluated LP-S’s ability to target tumor metastatic lymph nodes in mice. These mice had been injected with CAL27 cells in their left legs, and after three weeks, primary tumors had developed. We then injected LP-S (25ug/ml, 50ul) into the hind footpad and observed its migration to the tumor using wide field fluorescence imaging, shown in Fig. 6a. Within 30 min, LP-S traveled to both the tumor metastatic lymph nodes (T-LNs) and the normal lymph nodes (N-LNs), with higher accumulation of LP-S in the T-LNs than in the N-LNs (Fig. 6b). The fluorescence signal in T-LNs also decreased more slowly over a 3-hour period than in the N-LNs, as shown in Fig. 6d. However, after 5 h, the signal-to-background ratio (SBR) for both types of LNs decreased because of increasing fluorescence in normal tissues, making it difficult to tell them apart, as seen in Fig. 6e. Interestingly, 24 h after injection and mouse was sacrificed and the skin removed, the fluorescence intensity of LP-S in the T-LNs was still strong, clearly differentiating them from the N-LNs. When we looked at the resected LNs directly, we saw a significant difference in fluorescence intensity between T-LNs and N-LNs, shown in Fig. 6c. In summary, these results demonstrate that LP-S can effectively differentiate T-LNs from N-LNs based on its uptake and retention, especially during the first 3 h after injection.

### Fluorescence-guided surgery

**Fig. 7:**
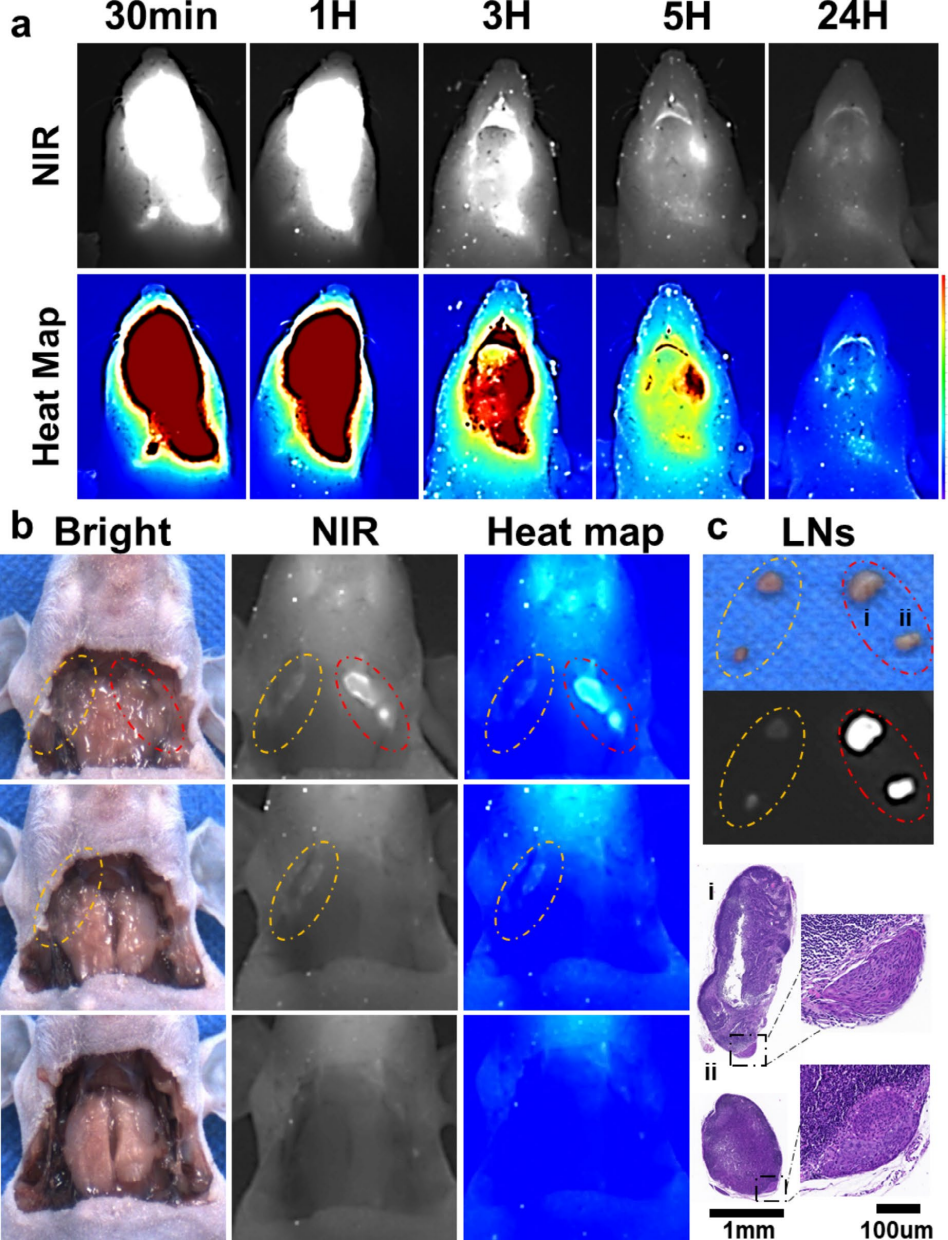
LP-S enables fluorescence-guided surgery for the excision of cervical mLNs in tongue cancer mice. (**a**) Representative NIR fluorescence images and heat maps of cervical lymphatic drainage at different time points after local intra-tumoral injection of LP-S (25ug/ml, 50ul). (**b**) Fluorescence-guided surgery for the excision of cervical LNs after 24 h injection, including highlighting (red circle) and contralateral (yellow circle) LNs. (**c**) Fluorescence images of excised cervical LNs after 24 h injection of LP-S. H&E staining of highlighted LNs confirmed metastasis

We further explored LP-S’s potential for fluorescence-guided surgery using a mouse model with in-situ tongue cancer and associated cervical metastatic lymph nodes, shown in Fig. 7. We injected LP-S directly into the primary tongue tumor (25ug/ml, 50ul) and then performed wide field fluorescence imaging, as shown in Fig. 7a. Encouragingly, just as with the lymph nodes in the hind limbs, we were able to quickly detect cervical lymph nodes within just 30 min. While the fluorescence intensity in the cervical lymph nodes decreased over time, the mLNs maintained a strong fluorescence signal.

At 24 h after injecting LP-S, we prepared for fluorescence-guided surgery by exposing the mouse’s neck area, which is shown in Fig. 7b. With the overlying skin removed, the cervical lymph nodes, especially the metastatic ones (mLNs), were clearly visible. We then surgically removed these illuminated cervical lymph nodes, which were confirmed to be metastatic, as illustrated in Fig. 7c. Following this, non-metastatic lymph nodes on the opposite side were also extracted using the guidance of fluorescence imaging. In total, across 6 mouse models, we removed 19 cervical lymph nodes, simulating real-world surgical scenarios. Out of these, 6 out of the 8 fluorescent lymph nodes were confirmed to contain metastases, as shown in Figure S6a. Remarkably, the false positive rate was only 25%, a significant improvement from previous clinical trials using ICG, which had a rate of 72.83%. Further validation outside the body (ex vivo) highlighted the stronger fluorescence intensity in mLNs compared to normal lymph nodes, as displayed in Figure S6b. An in-depth analysis of the Receiver Operating Characteristic (ROC) curve confirmed the accuracy of LP-S. The Area Under the Curve (AUC) was 0.9231, with a 95% confidence interval ranging from 0.7987 to 1.000, as presented in Figure S6c. These results demonstrate the accuracy of LP-S in identifying mLNs during fluorescence-guided surgery, and its potential to improve future surgical procedures.

## Discussion

The drainage pathways of cervical lymph nodes (LNs) in oral squamous cell carcinoma (OSCC) patients lack a standardized route. Previous studies propose that variations in sentinel LN (SLN) locations predominantly stem from disparities in the primary tumor’s position. Consequently, developing a new method for precise intraoperative identification of metastatic LNs is necessary to improve patient outcomes. Such an advancement will better enable surgeons to make informed treatment decisions that yield improved outcomes and mitigate complexities associated with LN removal.

Radiotracers, such as Tc99m-labeled colloids, and chromophores like methylene blue and nanocarbon, have been developed for LN detection [34–37]. However, each of these approaches carries its own set of considerations. The utilization of radioactive tracers introduces concerns about the detrimental effects of radiation exposure, while the visibility offered by chromophores often cannot penetrate tissue surfaces. Near-infrared (NIR) fluorescence imaging agents, offer an alternative approach to LN tracking without ionizing radiation and better tissue penetrance than visible light chromophores. In recent years, many innovative fluorescent probes that outperform ICG in different targeted applications with significant improvements in lymph node (LN) labeling and accurate resection for some cancer types [38–40].

In this work, we have presented a new method that combines a NIR fluorescent dye with the drug Lapatinib. This combination aids in tracking metastatic lymph nodes in cases of oral squamous cell carcinoma (OSCC) where there is an overexpression of the epidermal growth factor receptor (EGFR). Lapatinib, which inhibits both EGFR (HER1) and HER2, was selected due to its strong and specific binding to HER1 and HER2, with IC50 values of 10.2 nmol (HER1) and 9.8 nmol (HER2) [41–43]. Additionally, Lapatinib’s active aliphatic amino functional group offers a suitable site for conjugation with the carboxyl group, allowing easy drug modification.

When combined with the fluorescent molecule S0456, the resulting compound, LP-S, demonstrates notable binding to both HER1 and HER2, as confirmed by molecular docking and experimental results. We showed that LP-S selectively binds to OSCC cells with increased EGFR expression and undergoes internalization through EGFR-mediated endocytosis. This mechanism explains LP-S’s enhanced fluorescence intensity, improved contrast, and greater retention in mLNs compared to regular LNs.

Our findings indicate that when the fluorescent probe is coupled, it not only reduces the biological toxicity of Lapatinib but also maintains its effectiveness in EGFR-mediated endocytosis. While this surprising enhancement is promising, it necessitates a thorough examination to understand the exact mechanism behind it. One crucial concern when introducing new NIR fluorescent dyes for clinical use is their potential systemic toxicity. While decreased toxicity is a significant advantage for their future use as imaging agents, it also raises a challenge. Reducing toxicity to tumor cells might unintentionally weaken the effectiveness of cancer treatments. One possible solution to this potential problem is demonstrated by Fan et al. who have demonstrated a nanoprobe for both labeling and treating mLNs as part of the surgical removal procedure [44]. This combination of both diagnostic and treatment capabilities using multi-functional nanomaterials might represent a new strategy for treating mLNs.

Compared to nanoparticles, low molecular weight fluorescent probes can increase intracellular uptake. However, they also tend to spread into normal tissues, leading to background noise and decreasing Signal-to-Background Ratios (SBR) [45]. Despite this challenge, LP-S has shown remarkable optical performance, surpassing clinically approved ICG in tumor imaging and lymph node tracking. Compared to ICG, LP-S demonstrates a twenty fold increase in quantum yield resulting in stronger fluorescence signals. Furthermore, the larger size of LP-S reduces diffusion into surrounding normal tissues, reducing background noise. This combination of effects means tumors can be identified with lower doses of the probe and result in better contrast and longer observation times for LN imaged with LP-S making LP-S a significantly improved imaging agent relative to ICG.

Finally, LP-S overcomes the common issue of imaging agent not penetrating tissues deeply enough for clinical use. Our observations confirm that both metastatic and healthy LNs in the drainage area show high fluorescent contrast under direct imaging 24 h after local injection of LP-S with the metastatic LNs having significantly higher fluorescence allowing easy identification. Further, for tumor imaging, LP-S showed high contrast in all experiments at every combination of injection site and incubation time investigated. This combination of properties makes LP-S well suited for use in OSCC resection procedures and an attractive agent for future clinical development.

### Electronic supplementary material

Below is the link to the electronic supplementary material.

Supplementary Material 1

## Data Availability

The original contributions presented in the study are included in the article / supplementary information, further materials are available from contacting the corresponding author.
