# Supplementary material for: A novel near-infrared EGFR targeting probe for metastatic lymph node imaging in preclinical mouse models
Source: J Nanobiotechnology. 2023 Sep 22;21:342. doi: 10.1186/s12951-023-02101-z (PMC10514992; doi:10.1186/s12951-023-02101-z)
Supplement: Supplementary file 1 — Supplementary Material 1 [file 12951_2023_2101_MOESM1_ESM.docx]

A novel near-infrared EGFR targeting probe for metastatic lymph node imaging in preclinical mouse models

Diya Xie ^1,#^, Yunlong Li ^2,3,#^, Jiahong Shi ^4^, Yao ping Zhu ^1^, Yiqing Wang ^2,3,*^, Christopher J. Butch ^2,3,*^, Zhiyong Wang ^1,*^

1. Department of Oral and Maxillofacial Surgery, Nanjing Stomatological Hospital, Affiliated Hospital of Medical School, Nanjing University, Nanjing, China;

2.Department of Biomedical Engineering, College of Engineering and Applied Sciences, Nanjing University, Nanjing, China;

3.State Key Laboratory of Analytical Chemistry for Life Science, Nanjing University, Nanjing, China;

4. Department of Periodontics, Nanjing Stomatological Hospital, Affiliated Hospital of Medical School, Nanjing University, Nanjing, China.

**Supplementary Information**

**Materials and Methods**

Nuclear magnetic resonance (NMR) spectra, including ^1^H, ^13^C, were obtained using a Bruker 500 NMR spectrometer and signals are reported in ppm. We used the SHIMADU LC-2020 system to test compound purity. Analytical column type was SHIMADZU Inertsil ODS-SP(4.6*250mm*5um). For liquid chromatography-mass spectrometry (LC-MS), a Waters 2695 Alliance HPLC System coupled with a Waters 2998 diode array detector and a Waters 3100 SQ mass spectrometer was employed. Pump A was 0.1% trifluoroacetic in 100% water. Pump B was 0.1% trifluoroacetic in 100% acetonitrile. The flow rate was 1 ml/min with the solvent gradient starting from 75% solvent A (0.1% trifluoroacetic in 100% water) and 25% solvent B (0.1% trifluoroacetic in 100% acetonitrile) to 0% solvent A and 100% solvent B at 23 min. All the isolated compounds demonstrated ≥98% purity at λ = 220nm in the aforementioned analytical HPLC method. High-resolution mass spectrometry data were acquired using a Waters SYNAPT G2-Si high definition mass spectrometry.

**Quantum yield measurements**

Quantum yield (QY) of LP-S was determined in H_2_O with ICG (0.48%) that emited in the NIR window as the fluorescence reference standard provided that the excitation wavelength and emission wavelength of the reference and the analyte were similar. When the absorption value of LP-S and ICG solutions was 0.1, we measured the fluorescence emission curves of the respective solutions and calculate their integral areas. The calculation formula is:

QY_LP-S_ = {(F / A) / (F_0_ / A_0_)} * (f / f_0_) * QY_ICG_

QY_LP-S_=9.45%.

Q, F, A, f are the quantum yield, fluorescence integral area, absorbance and refractive index of the analyte, respectively.

**Cytotoxicity measurement**

The cytotoxicity measurement of the samples to OSCC cells was conducted by CCK-8 assay. Briefly, 1×10^4^ cells (100μl) were plated into each well of a 96 well culture plate and incubated in an atmosphere of 5% CO_2_ at 37℃ overnight. In each well, 100μl of fresh medium containing different concentrations of Lapatinib and LP-S (1nM-10uM) was used to replace the medium. Culture medium was aspirated after incubation for 24h and treated cells were washed two times with PBS. 90ul of fresh medium and 10μl of CCK-8 were then added to each well and continued for 2h incubation at 37°C. Then the absorbance of each well at 450 nm was measured by iMark Enzyme mark instrument (BIO-RAD Inc., USA). Cytotoxicity was calculated on the basis of absorbance.

**Western blot**

Cells were harvested and subjected to lysis in RIPA buffer. Lysed samples were assayed for protein concentration (NanoDrop One, ThermoFisher Scientific). An equal amount of protein (50 μg/lane) from the samples was loaded and separated by electrophoresis on an SDS-polyacrylamide gel electrophoresis and then transferred to an Immobilon-P membrane (Immobilon, Millipore Corporation, Bedford, MA). Membranes were blocked for western blotting using Tris buffered saline containing 5% skimmed milk powder and 0.1% Tween 20, followed by incubation with anti-EGFR antibody (Cat. No. ZRB04338, Sigma-Aldrich) at a ratio of 1:1000. The signals were detected by using an ECL system (Amersham, Piscataway, NJ).

**Results**


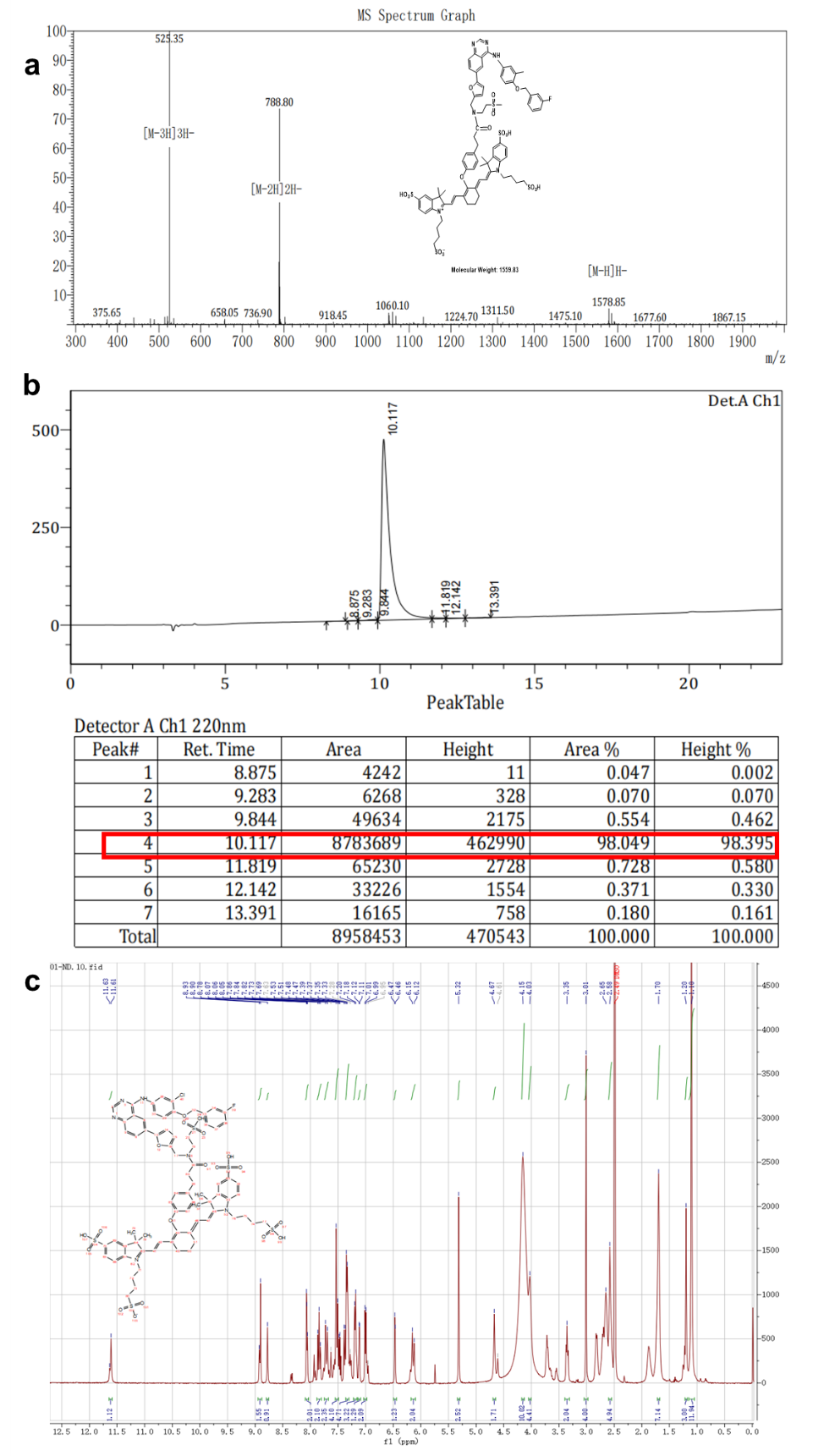


Fig.S1 Characterization of LP-S. a.MS spectrum of LP-S. b. High-resolution mass spectrometry data. c. 1H NMR of LP-S.


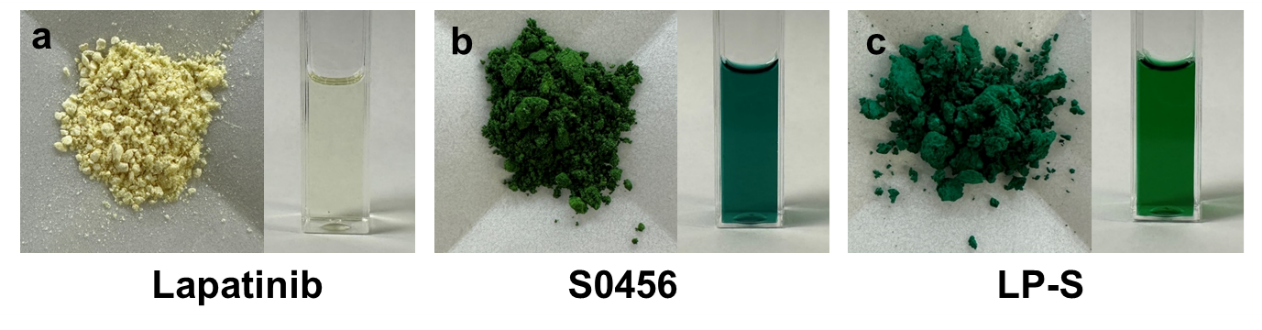


Fig.S2 LP-S exhibits good water solubility. Powder and water solution of Lapatinib (a), S0456 (b) and LP-S (c).


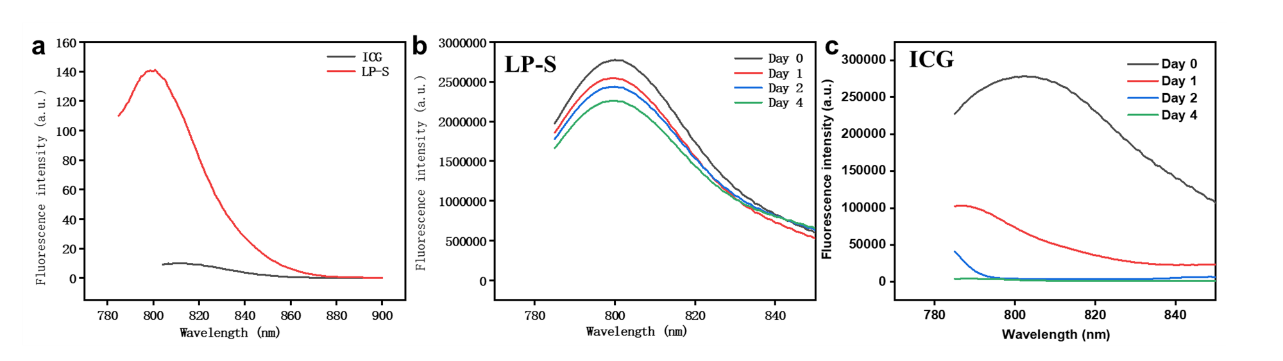


Fig.S3 The fluorescence emission curves of the LP-S and ICG solutions when their absorption value is 0.1. b. The fluorescence emission curves of LP-S (1nmol/L) at different times. c. The fluorescence emission curves of ICG (1nmol/L) at different times.


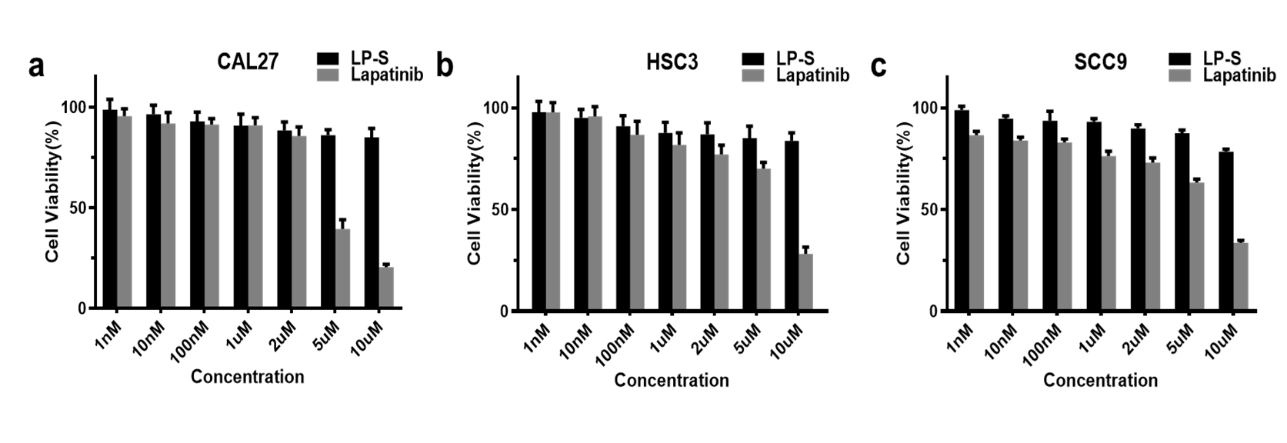


Fig.S4 OSCC cells viability after simple co-incubation with LP-S or Lapatinib (1nM-10uM). a. Cal27. b. HSC3. c. SCC9.


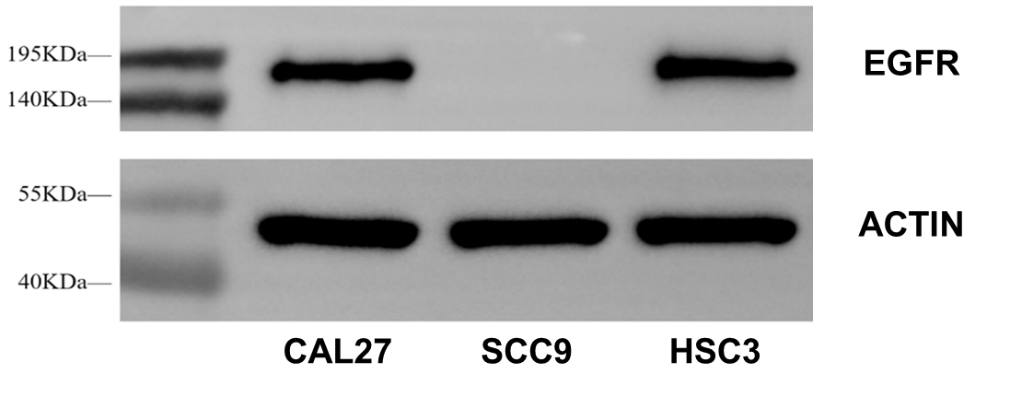


Fig.S5 The expression of EGFR in CAL 27, SCC9 and HSC3cells was determined by WB assay. Actin was chosen as the reference protein.


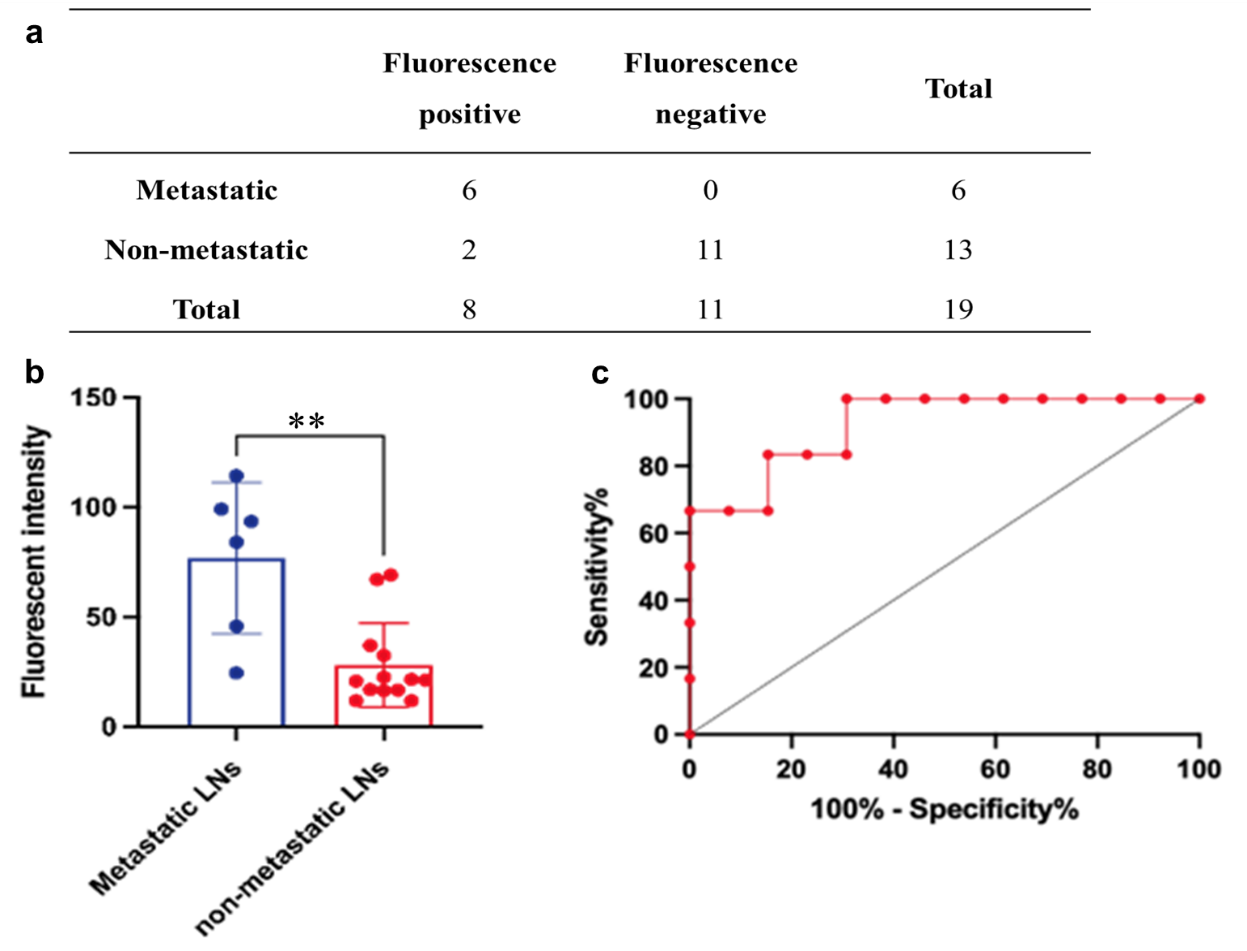


Fig.S6 Analysis of LNs excised by fluorescence-guided surgery. a. Fluorescence and pathological status of excised cervical lymph nodes in tongue cancer mice undergoing fluorescence-guided surgery with LP-S. b. Quantitative analysis of fluorescence intensities in metastatic and non-metastatic LNs. (** P< 0.01) c. ROC curve analysis showed the AUC is 0.9231 (95% confidence interval: 0.7987-1.000).
